# Supplementary material for: MetaRibo-Seq measures translation in microbiomes
Source: Nat Commun. 2020 Jun 29;11:3268. doi: 10.1038/s41467-020-17081-z (PMC7324362; doi:10.1038/s41467-020-17081-z)
Supplement: Supplementary file 10 — Supplementary Data 7 [file 41467_2020_17081_MOESM10_ESM.zip › File2/Confidence_High_Taxonomy/130468_out.krona.html]

Javascript must be enabled to view this page.

members
magnitude
magnitudeUnassigned
count
unassigned
taxon
rank

130468\_out

427

427
superkingdom
2

phylum
976
426

426
200643
class

171549
order
426

815
family
425


SRS013940\_contig\_number\_19370SRS015369\_contig\_number\_8963SRS015854\_contig\_number\_contig-100\_166.89462SRS015890\_contig\_number\_15736SRS019068\_contig\_number\_88561SRS019267\_contig\_number\_1969SRS019968\_contig\_number\_10876SRS021948\_contig\_number\_contig-100\_3350.181950SRS023346\_contig\_number\_4485SRS024625\_contig\_number\_11381SRS045004\_contig\_number\_15702SRS049900\_contig\_number\_14747SRS049995\_contig\_number\_13735SRS053356\_contig\_number\_47964SRS054352\_contig\_number\_contig-100\_1958.140742SRS057480\_contig\_number\_contig-100\_2812.70914SRS064557\_contig\_number\_contig-100\_2114.82519SRS075398\_contig\_number\_contig-100\_309.310SRS075963\_contig\_number\_contig-100\_1554.1554SRS105153\_contig\_number\_30700SRS140513\_contig\_number\_14883SRS143876\_contig\_number\_19986SRS147022\_contig\_number\_1973SRS147346\_contig\_number\_42522SRS148253\_contig\_number\_11347SRS893382\_contig\_number\_1025SRS971275\_contig\_number\_contig-100\_3176.252077
genus
425
816
27

species
457391

SRS043001\_contig\_number\_1326
1

species
2292944

SRS013800\_contig\_number\_3378SRS049900\_contig\_number\_1380SRS149325\_contig\_number\_7860
3

17

SRS015960\_contig\_number\_21365SRS016018\_contig\_number\_898SRS018049\_contig\_number\_276SRS018984\_contig\_number\_86SRS019286\_contig\_number\_3990SRS024663\_contig\_number\_3728SRS042628\_contig\_number\_29811SRS045195\_contig\_number\_6205SRS048981\_contig\_number\_2570SRS052078\_contig\_number\_3986SRS054590\_contig\_number\_237SRS064276\_contig\_number\_15918SRS101268\_contig\_number\_251SRS1041112\_contig\_number\_2565SRS1055067\_contig\_number\_10220SRS147139\_contig\_number\_3501SRS148874\_contig\_number\_1214
357276
species


SRS020394\_contig\_number\_14489SRS048060\_contig\_number\_12413SRS1054691\_contig\_number\_4704
3
species
2292935

1

SRS104912\_contig\_number\_21090
1263052
species

1

SRS017745\_contig\_number\_770
387090
species


SRS077086\_contig\_number\_4707SRS077231\_contig\_number\_6214SRS893378\_contig\_number\_556
3
species
1263056


SRS045244\_contig\_number\_2111SRS142923\_contig\_number\_contig-100\_415.137483
2
species
246787


SRS011239\_contig\_number\_contig-100\_422.133980SRS011302\_contig\_number\_28168SRS011405\_contig\_number\_14341SRS012902\_contig\_number\_155SRS013158\_contig\_number\_4235SRS013382\_contig\_number\_387SRS013521\_contig\_number\_9223SRS013638\_contig\_number\_10826SRS013965\_contig\_number\_37911SRS014287\_contig\_number\_1114SRS014313\_contig\_number\_754SRS014534\_contig\_number\_335SRS014683\_contig\_number\_18850SRS014736\_contig\_number\_853SRS014855\_contig\_number\_5373SRS014948\_contig\_number\_4309SRS014979\_contig\_number\_29596SRS015190\_contig\_number\_4448SRS015217\_contig\_number\_29039SRS015431\_contig\_number\_34395SRS015486\_contig\_number\_751SRS015579\_contig\_number\_19391SRS015663\_contig\_number\_21526SRS015694\_contig\_number\_4156SRS015782\_contig\_number\_29079SRS015794\_contig\_number\_7908SRS016056\_contig\_number\_2358SRS016095\_contig\_number\_23408SRS016132\_contig\_number\_692SRS016203\_contig\_number\_8502SRS016335\_contig\_number\_34193SRS016753\_contig\_number\_1826SRS016840\_contig\_number\_1327SRS016897\_contig\_number\_313SRS016954\_contig\_number\_16804SRS016989\_contig\_number\_6399SRS017103\_contig\_number\_33982SRS017191\_contig\_number\_11306SRS017247\_contig\_number\_5280SRS017307\_contig\_number\_18158SRS017375\_contig\_number\_224SRS017433\_contig\_number\_15392SRS017521\_contig\_number\_31188SRS017701\_contig\_number\_11886SRS017821\_contig\_number\_2764SRS018133\_contig\_number\_3404SRS018351\_contig\_number\_14512SRS018623\_contig\_number\_5728SRS018817\_contig\_number\_3407SRS018836\_contig\_number\_1436SRS018888\_contig\_number\_contig-100\_154.56539SRS018936\_contig\_number\_2541SRS019030\_contig\_number\_14877SRS019381\_contig\_number\_3244SRS019397\_contig\_number\_12585SRS019445\_contig\_number\_18076SRS019496\_contig\_number\_14944SRS019601\_contig\_number\_21757SRS019693\_contig\_number\_9117SRS020271\_contig\_number\_contig-100\_297.298SRS020622\_contig\_number\_6148SRS020869\_contig\_number\_24002SRS021153\_contig\_number\_1150SRS021484\_contig\_number\_19174SRS022071\_contig\_number\_35880SRS022137\_contig\_number\_18668SRS022524\_contig\_number\_15875SRS022713\_contig\_number\_746SRS023526\_contig\_number\_32542SRS023829\_contig\_number\_209SRS023971\_contig\_number\_2883SRS024009\_contig\_number\_7575SRS024331\_contig\_number\_30098SRS024492\_contig\_number\_16064SRS042284\_contig\_number\_1120SRS043667\_contig\_number\_2284SRS043701\_contig\_number\_13292SRS045645\_contig\_number\_14047SRS045739\_contig\_number\_3012SRS045826\_contig\_number\_15779SRS046068\_contig\_number\_2438SRS046369\_contig\_number\_7692SRS046502\_contig\_number\_10246SRS046810\_contig\_number\_contig-100\_73.74SRS047044\_contig\_number\_2306SRS047433\_contig\_number\_14356SRS048164\_contig\_number\_13896SRS049402\_contig\_number\_9323SRS049446\_contig\_number\_868SRS049712\_contig\_number\_426SRS049773\_contig\_number\_29021SRS049896\_contig\_number\_12705SRS049959\_contig\_number\_37664SRS050026\_contig\_number\_3105SRS050422\_contig\_number\_20870SRS050520\_contig\_number\_473SRS050752\_contig\_number\_23328SRS050925\_contig\_number\_25645SRS050974\_contig\_number\_1805SRS050998\_contig\_number\_15445SRS051031\_contig\_number\_30199SRS051610\_contig\_number\_9111SRS051882\_contig\_number\_10496SRS052576\_contig\_number\_407SRS052697\_contig\_number\_42958SRS053087\_contig\_number\_1575SRS053335\_contig\_number\_15450SRS053398\_contig\_number\_19865SRS054059\_contig\_number\_1043SRS054905\_contig\_number\_2150SRS055017\_contig\_number\_20104SRS055966\_contig\_number\_103SRS056273\_contig\_number\_27936SRS056537\_contig\_number\_34515SRS057478\_contig\_number\_1631SRS058070\_contig\_number\_20645SRS058268\_contig\_number\_22SRS058723\_contig\_number\_2098SRS058770\_contig\_number\_15287SRS062427\_contig\_number\_831SRS062654\_contig\_number\_2215SRS062701\_contig\_number\_1074SRS063127\_contig\_number\_210SRS063190\_contig\_number\_9339SRS063489\_contig\_number\_4176SRS063518\_contig\_number\_28880SRS063813\_contig\_number\_4989SRS063928\_contig\_number\_535SRS064557\_contig\_number\_3933SRS064757\_contig\_number\_21642SRS065397\_contig\_number\_11753SRS065504\_contig\_number\_10522SRS074586\_contig\_number\_4700SRS074670\_contig\_number\_3198SRS074964\_contig\_number\_694SRS075078\_contig\_number\_29169SRS075716\_contig\_number\_3995SRS075773\_contig\_number\_26711SRS075821\_contig\_number\_999SRS075878\_contig\_number\_16138SRS076756\_contig\_number\_18627SRS076976\_contig\_number\_13808SRS077194\_contig\_number\_12505SRS077294\_contig\_number\_18148SRS077335\_contig\_number\_1872SRS077392\_contig\_number\_40518SRS077454\_contig\_number\_9744SRS077502\_contig\_number\_2610SRS077641\_contig\_number\_17691SRS077849\_contig\_number\_25947SRS077991\_contig\_number\_255SRS078176\_contig\_number\_18374SRS078242\_contig\_number\_19199SRS078419\_contig\_number\_9587SRS078665\_contig\_number\_7530SRS097889\_contig\_number\_24011SRS097920\_contig\_number\_11358SRS097958\_contig\_number\_14299SRS098061\_contig\_number\_12279SRS098571\_contig\_number\_16375SRS098644\_contig\_number\_30878SRS098655\_contig\_number\_8351SRS098827\_contig\_number\_2312SRS098881\_contig\_number\_14929SRS100907\_contig\_number\_8800SRS101376\_contig\_number\_24546SRS101433\_contig\_number\_12486SRS103987\_contig\_number\_4196SRS104036\_contig\_number\_6404SRS104084\_contig\_number\_13093SRS1041031\_contig\_number\_27250SRS1041036\_contig\_number\_4448SRS1041037\_contig\_number\_13796SRS1041038\_contig\_number\_16920SRS1041090\_contig\_number\_5562SRS1041091\_contig\_number\_8226SRS1041116\_contig\_number\_9206SRS1041118\_contig\_number\_484SRS1041130\_contig\_number\_2531SRS1041133\_contig\_number\_24106SRS1041134\_contig\_number\_10153SRS1041136\_contig\_number\_18304SRS1041137\_contig\_number\_12377SRS1041139\_contig\_number\_2377SRS1041140\_contig\_number\_21368SRS1041141\_contig\_number\_13095SRS1041142\_contig\_number\_1355SRS1041143\_contig\_number\_1579SRS1041144\_contig\_number\_7636SRS1041145\_contig\_number\_27188SRS1041147\_contig\_number\_2030SRS1041157\_contig\_number\_5233SRS1041164\_contig\_number\_1470SRS104327\_contig\_number\_6152SRS104400\_contig\_number\_57178SRS104485\_contig\_number\_15SRS104975\_contig\_number\_1771SRS105082\_contig\_number\_488SRS1054716\_contig\_number\_14868SRS1054928\_contig\_number\_4222SRS1055034\_contig\_number\_11768SRS1055038\_contig\_number\_1809SRS1055049\_contig\_number\_15309SRS1055076\_contig\_number\_15223SRS1055083\_contig\_number\_9489SRS1055095\_contig\_number\_784SRS140492\_contig\_number\_30248SRS142503\_contig\_number\_17582SRS142542\_contig\_number\_19371SRS142599\_contig\_number\_78SRS142890\_contig\_number\_24267SRS142980\_contig\_number\_1330SRS143070\_contig\_number\_15080SRS143085\_contig\_number\_4975SRS143148\_contig\_number\_13458SRS143181\_contig\_number\_14003SRS143342\_contig\_number\_6712SRS143372\_contig\_number\_7229SRS143523\_contig\_number\_22387SRS143722\_contig\_number\_8371SRS143895\_contig\_number\_17893SRS143991\_contig\_number\_14100SRS144135\_contig\_number\_31121SRS144183\_contig\_number\_35628SRS144297\_contig\_number\_8006SRS144362\_contig\_number\_5214SRS144537\_contig\_number\_18623SRS144603\_contig\_number\_7135SRS144714\_contig\_number\_12383SRS145497\_contig\_number\_12356SRS146764\_contig\_number\_17106SRS146812\_contig\_number\_38973SRS147039\_contig\_number\_1565SRS147271\_contig\_number\_15516SRS147377\_contig\_number\_10921SRS147425\_contig\_number\_5733SRS147653\_contig\_number\_7444SRS147766\_contig\_number\_13944SRS147977\_contig\_number\_10203SRS148091\_contig\_number\_13368SRS148159\_contig\_number\_24159SRS148196\_contig\_number\_34075SRS148424\_contig\_number\_27627SRS148721\_contig\_number\_54023SRS148817\_contig\_number\_11928SRS148970\_contig\_number\_3588SRS149075\_contig\_number\_10969SRS149181\_contig\_number\_17646SRS149244\_contig\_number\_2009SRS150029\_contig\_number\_14439SRS892882\_contig\_number\_14863SRS893170\_contig\_number\_8168SRS893230\_contig\_number\_8091SRS893252\_contig\_number\_13288SRS893253\_contig\_number\_557SRS893259\_contig\_number\_5309SRS893270\_contig\_number\_24731SRS893279\_contig\_number\_15135SRS893292\_contig\_number\_6889SRS893295\_contig\_number\_12235SRS893300\_contig\_number\_8993SRS893327\_contig\_number\_3346SRS893341\_contig\_number\_10999SRS893342\_contig\_number\_8677SRS893358\_contig\_number\_9598SRS893366\_contig\_number\_17930SRS893369\_contig\_number\_8734SRS893373\_contig\_number\_26125SRS893380\_contig\_number\_885SRS971276\_contig\_number\_32827
270
species
821


SRS013215\_contig\_number\_1206SRS014613\_contig\_number\_4295SRS023914\_contig\_number\_14438SRS050299\_contig\_number\_9192SRS057717\_contig\_number\_2242SRS077552\_contig\_number\_3086SRS1055022\_contig\_number\_3803SRS143780\_contig\_number\_1069SRS893285\_contig\_number\_784
9
species
818

1263042
species
7

SRS013476\_contig\_number\_39769SRS018541\_contig\_number\_12712SRS023715\_contig\_number\_13306SRS024549\_contig\_number\_449SRS075341\_contig\_number\_12370SRS142712\_contig\_number\_5126SRS149784\_contig\_number\_26955

47678
species
4

SRS019685\_contig\_number\_21668SRS024132\_contig\_number\_34610SRS077024\_contig\_number\_7314SRS143598\_contig\_number\_1797


SRS064973\_contig\_number\_38SRS075984\_contig\_number\_21059SRS143417\_contig\_number\_contig-100\_300.162874
3
species
28116


SRS018575\_contig\_number\_1216
1
species
376806

671267
species
37

SRS012969\_contig\_number\_34190SRS013951\_contig\_number\_38516SRS014235\_contig\_number\_18010SRS014923\_contig\_number\_41858SRS015264\_contig\_number\_20121SRS016495\_contig\_number\_18226SRS018427\_contig\_number\_32303SRS019161\_contig\_number\_9316SRS019910\_contig\_number\_7455SRS024388\_contig\_number\_10024SRS024435\_contig\_number\_40909SRS042966\_contig\_number\_17392SRS043768\_contig\_number\_5208SRS044535\_contig\_number\_11395SRS045713\_contig\_number\_19812SRS048870\_contig\_number\_17426SRS050801\_contig\_number\_3830SRS050941\_contig\_number\_13041SRS052027\_contig\_number\_5555SRS053214\_contig\_number\_13831SRS056259\_contig\_number\_23204SRS064232\_contig\_number\_4072SRS065176\_contig\_number\_6503SRS077127\_contig\_number\_14554SRS098073\_contig\_number\_17598SRS1041039\_contig\_number\_26223SRS1041092\_contig\_number\_4161SRS1041132\_contig\_number\_2402SRS1055056\_contig\_number\_13646SRS1055069\_contig\_number\_3432SRS144506\_contig\_number\_54337SRS144753\_contig\_number\_contig-100\_57.87418SRS147557\_contig\_number\_34150SRS147614\_contig\_number\_7591SRS148319\_contig\_number\_4783SRS148511\_contig\_number\_17831SRS893383\_contig\_number\_30238

817
species
8

SRS013098\_contig\_number\_25668SRS014459\_contig\_number\_19726SRS023583\_contig\_number\_17036SRS077730\_contig\_number\_1454SRS1041129\_contig\_number\_1019SRS1055043\_contig\_number\_25382SRS1055099\_contig\_number\_120SRS140645\_contig\_number\_266


SRS011084\_contig\_number\_11940
1
species
2292284

1

SRS893327\_contig\_number\_2294
1946087
species

1

SRS016381\_contig\_number\_2780
2292923
species

6

SRS021219\_contig\_number\_5376SRS022609\_contig\_number\_22649SRS063370\_contig\_number\_11910SRS064645\_contig\_number\_122SRS100021\_contig\_number\_8093SRS149879\_contig\_number\_11878
338188
species

674529
species
1

SRS063985\_contig\_number\_17690


SRS012273\_contig\_number\_12065SRS014412\_contig\_number\_6151SRS017859\_contig\_number\_4756SRS019178\_contig\_number\_1045SRS046717\_contig\_number\_6894SRS076876\_contig\_number\_141SRS077589\_contig\_number\_1884SRS146813\_contig\_number\_985SRS148784\_contig\_number\_25211SRS893288\_contig\_number\_733
10
species
46506

29523
species
1

SRS015065\_contig\_number\_22890

329854
species
1

SRS104636\_contig\_number\_1489

species
457395

SRS056519\_contig\_number\_731
1

3

SRS020508\_contig\_number\_540SRS098717\_contig\_number\_2539SRS145308\_contig\_number\_1093
1739517
species


SRS104693\_contig\_number\_8575
1
species
2292947

1

SRS011134\_contig\_number\_40918
162156
species

1
171552
family

577309
genus
1

1263095
species
1

SRS053649\_contig\_number\_11801

1
1239
phylum

class
186801
1

186802
order
1

31979
family
1

1485
genus
1

species
59620

SRS024075\_contig\_number\_12682
1
